# A 16-year retrospective investigation of granulomatous liver injury in immunocompetent and immunocompromised patients at a tertiary care center

**DOI:** 10.1093/ajcp/aqag114

**Published:** 2026-11-05

**Authors:** Charles H Wang, Kathleen Byrnes, Gillian L Hale

**Affiliations:** Department of Pathology, University of Utah, Salt Lake City, UT, United States; Department of Pathology and Immunology, Washington University in St Louis, St Louis, MO, United States; Department of Pathology, University of Utah, Salt Lake City, UT, United States

**Keywords:** hepatic granuloma, granulomatous hepatitis, molecular diagnostics, immunocompromised, infectious disease, sarcoidosis

## Abstract

**Objectives:**

Recent data on the underlying causes of granulomatous liver injury in the United States remain limited. In this study, we provide updated tertiary care center data on the causes of hepatic granulomas and evaluate the use of histochemical and molecular diagnostics to identify infectious organisms.

**Methods:**

We retrospectively reviewed 251 cases of hepatic granulomas (2009-2024) stratified by patient immune status. Additionally, we evaluated 6-year retrospective data from 2 institutions to determine the yield of broad-range molecular testing for infectious diseases.

**Results:**

Causes of the 251 cases included idiopathic (31%), autoimmune disease (20%), steatohepatitis (19%), sarcoidosis (10%), infection (8%), and drug reaction (4%). A subset (9%) of idiopathic cases showed features highly suspicious for infection, but standard infectious stains were unrevealing, and none were initially sent for molecular testing. Retrospective evaluation of send-out pathogen molecular testing across both institutions revealed organism detection in 32% (12/37) and 50% (4/8) of highly suspicious cases, respectively. Increasing clinicopathologic suspicion correlated positively with successful molecular pathogen identification and inversely with false-positive contamination rates.

**Conclusions:**

Molecular testing is frequently underused by pathologists in the workup of granulomatous hepatitis. Pathologists should proactively leverage molecular diagnostics to optimize clinical decision-making when standard histochemical stains are unrevealing.

Key points

- This study provides an update to decades-old data on hepatic granuloma causes and evaluates the modern utility of ­histochemical and molecular diagnostic workflows.
- Nine percent of idiopathic cases had features highly suspicious for infection, where broad-range molecular testing could have identified elusive pathogens.
- Pathologists frequently underuse send-out molecular testing, which has a high diagnostic yield for identifying pathogens when standard histochemical stains are negative.

## INTRODUCTION

Granulomas are dynamic cellular aggregates that comprise, at a minimum, histiocytes rimmed by lymphocytes and fibroblasts, formed in response to persistent stimuli or as a result of immune dysfunction.^1^ In the liver, granuloma formation can be evoked by a variety of insults, including infection, adverse drug reaction, systemic diseases like sarcoidosis, and autoimmune diseases such as primary biliary cholangitis. The histologic features of the granuloma (ie, size, shape, distribution, relative abundance, presence or absence of necrosis), together with histochemical stains, can help refine the differential diagnosis.^2‐4^ For example, sarcoidosis granulomas are typically lobular, well formed, epithelioid, and noncaseating, whereas primary biliary cholangitis granulomas are portal based, duct-centric, poorly formed, and rich in lymphoplasmacytic inflammation (ie, the florid duct lesion). Necrotizing granulomas can suggest the presence of mycobacterial and fungal infections. Ancillary testing, such as culture and serology, can aid in determining a specific diagnosis. However, the systemic manifestations of infection mimic those of neoplasia, and when infection is unsuspected, fresh tissue for culture studies may be unavailable. Pathologists play a critical role in the tissue-based diagnosis of infection, but overlapping inflammatory patterns, low sensitivity of special stains, and limited immunohistochemical staining panels challenge efforts at organism detection. Most cases of hepatic granulomas are attributed to sarcoidosis (22%) in the United States, autoimmune diseases (15.0-48.6%) in Europe, and tuberculosis (up to 88.9%) worldwide, while the cause remains unknown in 13% to 50% of cases worldwide.^4‐9^ The literature on the causes of granulomatous liver disease are decades-old, however, particularly those studies from the United States.^2^^,^^9^^,^^10^ As a major transplant center and academic institution that evaluates more than 500 liver biopsy specimens and liver explants annually, we sought to retrospectively evaluate the cause of granulomatous liver injury in all cases, assessing how causes differed according to patient immune status and the efficacy of ancillary histochemical tests in detecting infection. Correspondingly, we evaluated the utilization of send-out molecular testing for infectious causes in our granulomatous hepatitis cohort and, more generally, how our institution and another tertiary care institution used molecular infectious disease testing.

## METHODS

A retrospective 16-year (2009-2024) search of the electronic medical record was performed at the University of Utah using all possible pairings between 2 sets of keywords: [“granuloma,” “lymphohistiocytic”] and [“liver,” “portal,” “transplant,” “explant,” “hepatic,” “hepatitis,” “hepatectomy,” “resection”]. Duplicate entries (including cases where the same patient underwent repeat biopsies within 1 year) were removed, and only in-house cases that demonstrated definite liver granulomas were included in the final dataset. Data were extracted from each case, including patient demographic information, immune status, pathologic features of liver injury (ie, granuloma characteristics such as relative abundance, distribution, and presence of necrosis), features of the background liver, and all histochemical stain and ancillary test results. Detailed chart reviews were performed with clinicopathologic correlation to determine the cause of granulomatous liver injury. Patients were classified as immunocompromised if they were taking immunosuppressive medications (ie, high-dose corticosteroids, chemotherapeutics, medications for rheumatic diseases and allograft rejection) or had an immunosuppressive disease (common variable immune deficiency, AIDS, hemophagocytic lymphohistiocytosis).

All cases in which the cause of hepatic granulomata remained unknown and all cases clinically assumed to represent sarcoidosis were rereviewed histologically and restained for hematoxylin and eosin, acid-fast bacilli (AFB), Grocott’s methenamine silver (GMS), and a *Mycobacterium tuberculosis* immunohistochemical stain (Biocare, predilute antibody PP140 AA, performed on Leica Bond III). The causes of granulomata were compared between immunocompromised and immunocompetent patient subgroups.

Additionally, a retrospective 6-year (2019-2024) query was performed at the University of Utah to evaluate all specimens sent to an outside reference laboratory for panbacterial and fungal molecular testing. For each case, we evaluated the indication for molecular testing and test results, including whether genus- and species-level detection was reported, and assessed the relevance and implications of the results (ie, whether they represented true infections, contaminants, or false negatives) based on clinicopathologic correlation. We compared our findings with data from another large tertiary care institution (Washington University in St Louis), which performed a similar retrospective 6-year (2017-2022) evaluation of hepatic granuloma cases that underwent send-out pathogen molecular testing.

## RESULTS

### Causes and demographics of hepatic granulomas

Of 8503 liver specimens evaluated at the University of Utah from 2009 to 2024, 251 cases of liver granuloma were identified in 75 immunocompromised (30%) and 176 immunocompetent (70%) patients (**Table 1**). There were 217 core needle biopsy specimens, 24 subtotal hepatic excisions, and 10 hepatic explants. The patients’ ages ranged from 21 to 87 years, with a median age of 54 years; 150 patients were women, and 101 were men.

**Table 1:** Causes of Granulomas in the Liver^a^

Cause	Cases (n = 251), No. (%)	Immunocompromised (n = 75), No. (%)	Immunocompetent (n = 176), No. (%)	Diagnoses affected by pathology, No. (%)
Idiopathic	77 (31)	18 (24)	59 (34)	73 (95)
Autoimmune disease^b^	50 (20)	10 (13)	40 (23)	46 (92)
Steatohepatitis	48 (19)	17 (23)	31 (18)	48 (100)
Sarcoidosis	24 (10)	7 (9)	17 (10)	9 (38)
Infection	20 (8)	7 (9)	13 (7)	9 (45)
Drug induced	10 (4)	3 (4)	7 (4)	6 (60)
Foreign body	8 (3)	2 (3)	6 (3)	8 (100)
Allograft rejection	7 (3)	7 (9)	0 (0.0)	7 (100)
Malignancy	4 (2)	1 (1)	3 (2)	4 (100)
CVID	2 (1)	2 (3)	0 (0)	1 (50)
PSC	1 (0.4)	1 (1)	0 (0)	0 (0)

Abbreviations: CVID, common variable immune deficiency; PSC, primary sclerosing cholangitis.

a The 251 cases of liver granulomas were stratified by cause and patient immune status. We also identified cases in which the pathologic findings were essential for establishing a new diagnosis (in some cases, the diagnosis was unsuspected clinically; in others, histologic findings were needed to confirm a diagnosis or resolve the clinical differential).

b Autoimmune diseases included autoimmune hepatitis (AIH), primary biliary cholangitis (PBC), PBC-AIH overlap syndrome, and mixed connective tissue disease.

#### Idiopathic cases

If the cause of a liver granuloma could not be attributed to a single working diagnosis clinically or morphologically, despite detailed chart review, it was deemed to be idiopathic. In 77 of 251 cases, the granulomata were of unknown cause. Idiopathic cases comprised 24.0% of immunocompromised patients and 33.5% among immunocompetent patients. Seven (9%) idiopathic cases showed features highly suspicious for infection, including prominent neutrophilic or eosinophilic infiltrates, large confluent necrotizing granulomas, ill-defined granulomas with sinusoidal infiltration, and vasculitis (see **Figure 1** and **Table 2**).

**Figure 1:**
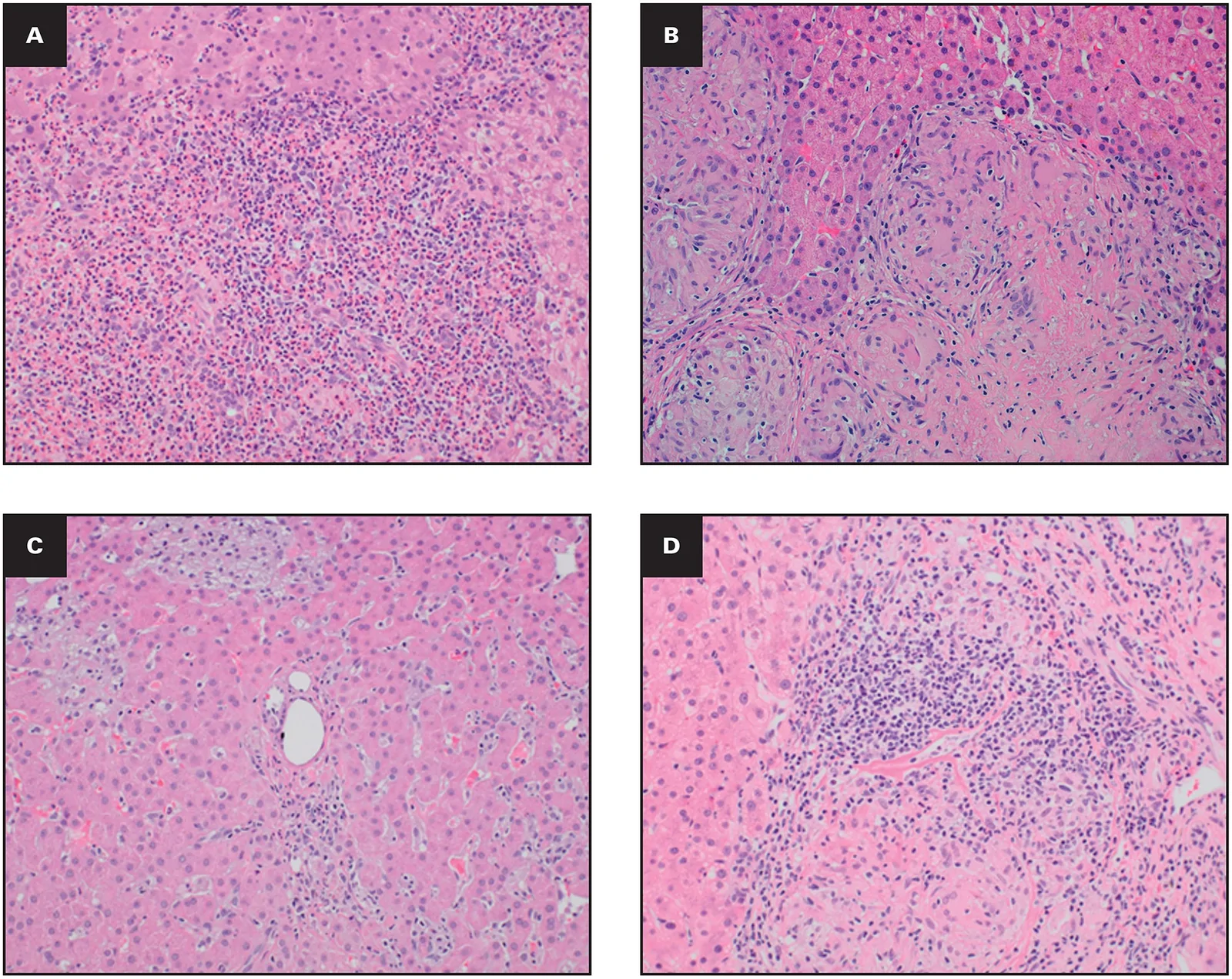
Morphologic features of cases suspicious for infection after the exclusion of other possibilities but for which no cause was determined. All panels stained with hematoxylin and eosin. (A) Prominent neutrophilic and admixed eosinophilic inflammation with hepatocyte injury. (B) Large confluent necrotizing granulomas. (C) Ill-defined granulomas with sinusoidal lymphohistiocytic infiltration. (D) Vasculitis.

**Table 2:** Idiopathic Cases That Remained Highly Suspicious for Infection^a^

Case No.	Feature suspicious for infection	Clinical history	Ancillary pathology testing	Follow-up
1	Eosinophilic infiltrates with necrosis	59-year-old woman with severe eosinophilia and a “diffuse process of the liver of uncertain etiology.” Multiple stable pulmonary nodules were noted 2 years prior, thought to represent granulomata from remote histoplasmosis due to avian exposure.	AFB (–) GMS (–) Gram (–) PAS (–)	No documentation.
2	Eosinophilic infiltrates with necrosis	56-year-old man with coronary artery disease and an incidental 2.5-cm liver mass.	AFB (–) GMS (–)	No further workup of liver granulomas. No other signs/symptoms of infection 12 years after liver biopsy.
3	Eosinophilic infiltrates with necrosis	71-year-old man with esophageal stricture and several liver lesions noted during esophagectomy.	AFB (–) GMS (–)	No further workup of liver granulomas. No other signs/symptoms of infection 9 months after liver biopsy.
4	Confluent necrosis	36-year-old man admitted for acute trauma, with incidental pulmonary, hepatic, and splenic nodules.	AFB (–) GMS (–) PAS (–) *T pall* (–)	Patient lost to follow-up 1 month after liver biopsy.
5	Sinusoidal lymphohistiocytic infiltration	42-year-old man with prior *Mycobacterium avium* complex infection, lymphadenopathy, and evidence of biliary obstruction.	AFB (–) PAS (–)	No documentation. Patient died 3 months after liver biopsy.
6	Vasculitis	79-year-old woman with decompensated cirrhosis and hepatocellular carcinoma. Paracentesis showed no definitive evidence of spontaneous bacterial peritonitis, and empiric antibiotics were discontinued.	AFB (–) GMS (–) PAS (–)	Patient died 4 months after liver biopsy and hospital discharge with no signs or symptoms of infection.
7	Numerous granulomas with no pulmonary involvement. Mycobacterial infection was not ruled out by laboratory testing.	71-year-old woman with diffuse large B-cell lymphoma who had recently completed chemotherapy, with concern for persistent lymphoma on imaging.	AFB (–) GMS (–)	Patient was diagnosed with sarcoidosis 2 months after liver biopsy, but imaging 2 years later showed spontaneous resolution of hepatic and splenic lesions, making sarcoidosis unlikely.

Abbreviations: AFB, acid-fast bacilli; GMS, Grocott’s methenamine silver; PAS, periodic acid–Schiff; *T pall*, *Treponema pallidum*; +, positive; –, negative.

a Of 77 cases of idiopathic liver granulomas, 7 were highly suspicious for infection based on histopathologic and clinical findings.

#### Autoimmune cases

In 50 of 251 cases (20%), the liver granulomas were attributed to autoimmune disease; 42 (84%) were primary biliary cholangitis. Histologically, most cases demonstrated classic portal-based nonnecrotizing granulomatous injury with a lymphohistiocytic infiltrate (ie, florid duct lesion). All cases had supportive clinical evidence of primary biliary cholangitis, including elevated antimitochondrial antibodies, antinuclear antibodies, and/or alkaline phosphatase.

#### Sarcoidosis cases

In 24 of 251 cases (10%), the liver granulomas were attributed to sarcoidosis (12% if excluding lipogranulomas). All sarcoidosis cases were rereviewed, revealing no evidence of infection by histochemical stains (AFB, GMS, and *M tuberculosis*). Morphologically, most cases showed multiple noncaseating well-formed lobular granulomas, consistent with sarcoidosis. One case demonstrated a single nonnecrotizing poorly formed portal granuloma in a patient with a self-reported history of pulmonary sarcoidosis who was treated with prednisone for 6 months but discontinued the therapy because of adverse effects. Three cases showed classic histologic findings for sarcoidosis but without evidence of pulmonary disease; these patients did not receive treatment but continued to carry diagnoses of sarcoidosis in their patient charts. In most cases, patients already had known, well-established diagnoses of sarcoidosis at the time of liver sampling. However, in 2 cases, pulmonary disease was discovered only after incidental liver granulomas were noted, resulting in new diagnoses of sarcoidosis.

#### Infectious cases

Infectious agents were identified in 20 of 251 (8%) liver granuloma cases (see **Table 3**). Infection was seen in 9% of immunocompromised patients and 7% of immunocompetent patients. The most frequent agent identified was hepatitis C virus, occurring in 1 immunocompromised patient and 5 immunocompetent patients, all diagnosed by serology. One case of hepatitis B virus was also diagnosed by serology. One case of cytomegalovirus showed viral inclusions histologically, with subsequent agreement by quantitative polymerase chain reaction for viral load. One case of Epstein-Barr virus, positive by in situ hybridization, was identified in the clinical setting of hemophagocytic lymphohistiocytosis, resulting in an exuberant inflammatory response in the liver with lobular granulomas and pericentral necrosis (see **Figure 2A, B**). Two cases of nontuberculous mycobacteria were detected in immunocompromised patients, both with AIDS; only 1 case showed acid-fast organisms histologically. One case of *Treponema pallidum* was found in an immunocompromised patient, confirmed by immunohistochemistry (see **Figure 2C, D**). One case of *Staphylococcus aureus* showed gram-positive cocci histologically, with confirmation of the organism by culture. Two cases of *Coxiella burnetii* were found in immunocompetent patients; 1 case was diagnosed clinically by serology, and the other was unsuspected clinically but showed fibrin ring granulomas histologically with supportive serologic test results (see **Figure 2E, F**). One case of *Candida albicans* resulted in necrotizing liver granulomas, with GMS stain showing pseudohyphae and yeast forms, and subsequent molecular testing identified the fungal species (see **Figure 2G, H**). Two cases of *Histoplasma capsulatum* were detected via urine antigen tests; subsequent histology identified a yeast form on the GMS stain in 1 case. One case of schistosomiasis was diagnosed without morphologic or serologic evidence of the parasite; rather, empiric treatment was suggested by the infectious disease team based on the patient’s travel history (Morocco, Italy, Ecuador, Bahamas, Mexico), given the presence of liver granulomas. One case of *Echinococcus* demonstrated refractile protoscoleces histologically.

**Figure 2:**
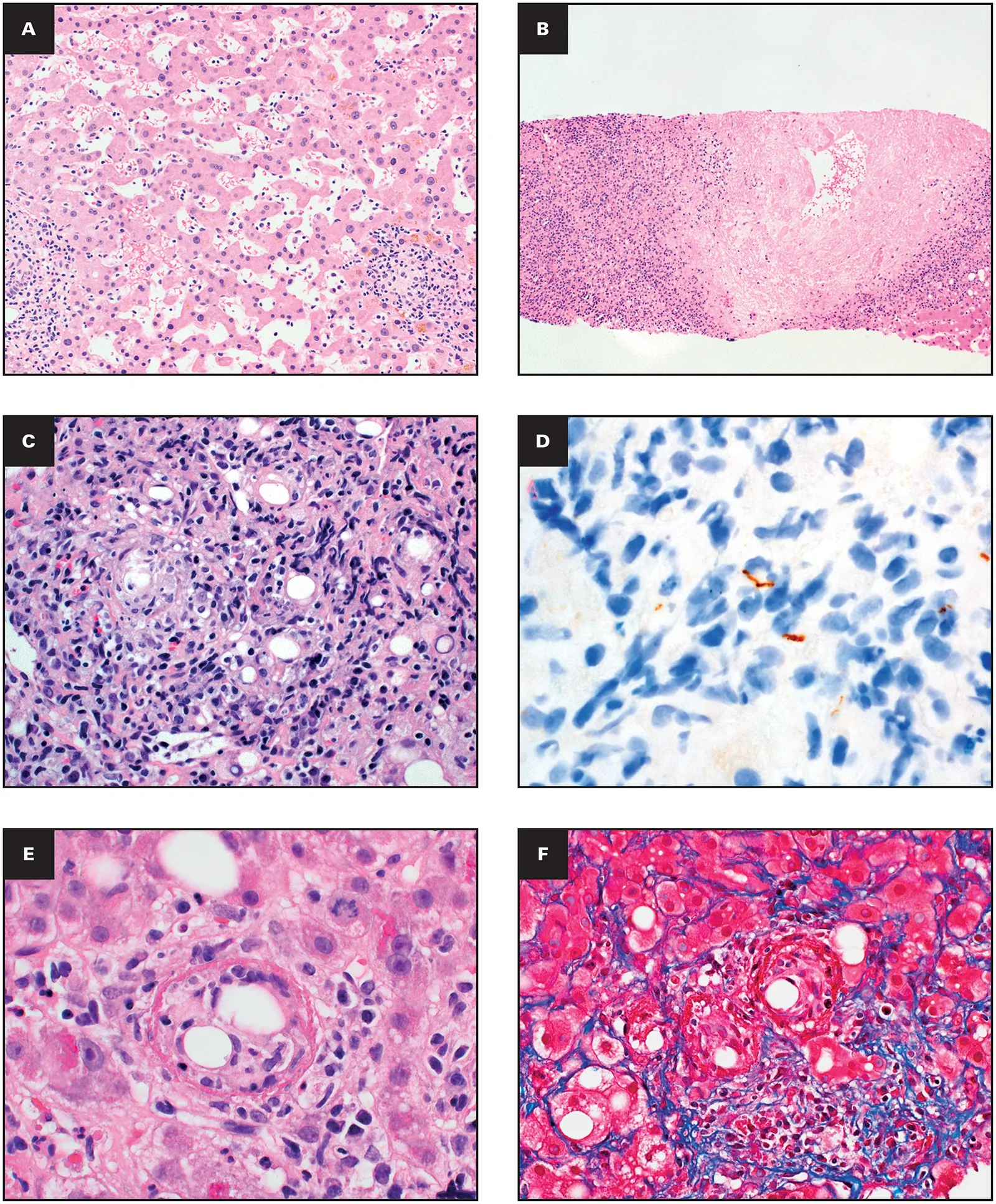
Histology of granulomatous hepatitis with known infectious causes. (A, B) Epstein-Barr virus hepatitis in the setting of hemophagocytic lymphohistiocytosis; hematoxylin and eosin (H&E) stain showing (A) lobular granulomas and (B) pericentral necrosis. (C, D) Syphilitic hepatitis on (C) H&E stain with corresponding spirochetes highlighted by (D) *Treponema pallidum* stain. (E, F) *Coxiella burnetii* infection demonstrating classic fibrin ring granulomas on (E) H&E stain. Fibrin ring granulomas are characterized by central lipid vacuoles surrounded by epithelioid macrophages, which are then encircled by rings of fibrin that stain deep red with a (F) trichrome stain.

**Figure 2:**
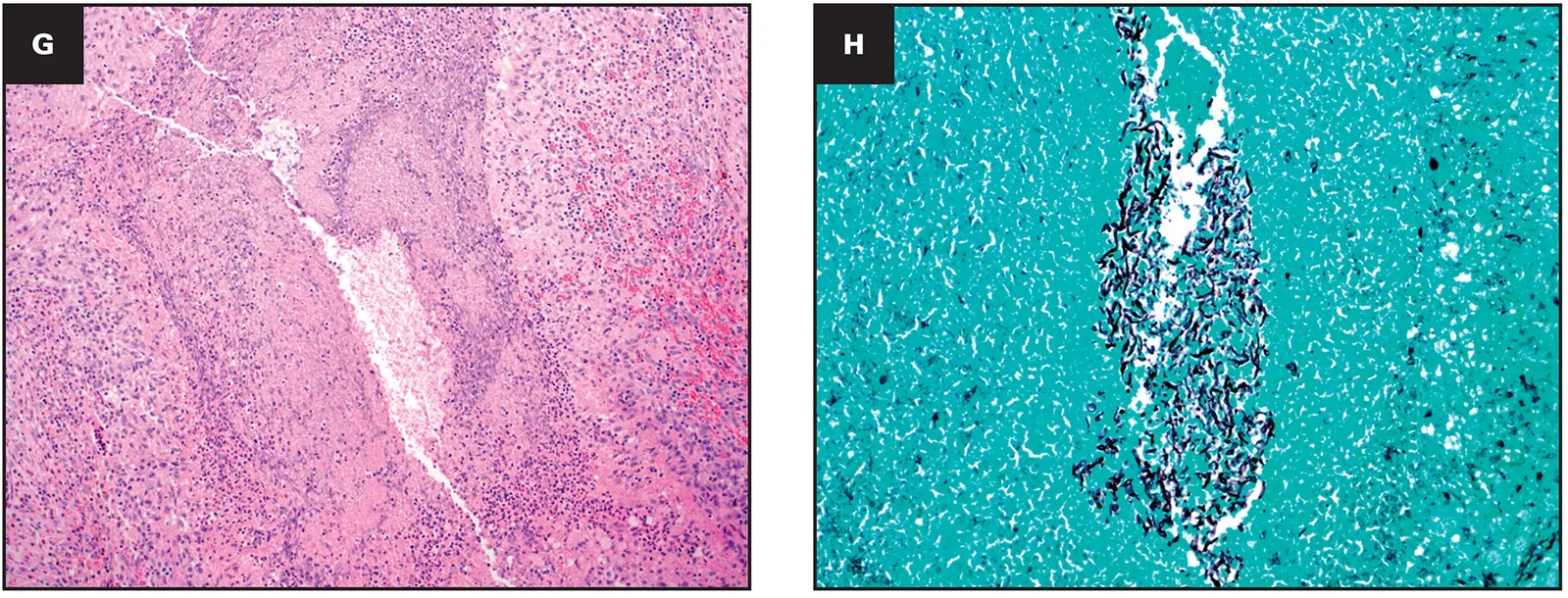
Continued (G, H) *Candida albicans* infection causing necrotizing liver granulomas on (G) H&E stain, with a Grocott methenamine silver (GMS) stain (H) clearly delineating hyphae and yeast forms.

**Table 3:** Pathogens Identified in Cases of Hepatic Granulomas^a^

Immunocompromised	Immunocompetent
Hepatitis C virus	Hepatitis C virus × 5
Cytomegalovirus	Hepatitis B virus
Epstein-Barr virus	
Nontuberculous mycobacteria	*Staphylococcus aureus*
*Mycobacterium avium* complex	*Coxiella burnetti* × 2
*Treponema pallidum*	
*Candida albicans*	*Histoplasma capsulatum* × 2
*Schistosoma mansoni*	*Echinococcus*

a We identified 20 infectious agents consisting of viruses, bacteria, fungi, and parasites in both immunocompromised and immunocompetent patients.

#### Drug-induced cases

In 10 of 251 cases (4%), the liver granulomas were attributed to an adverse drug reaction. For these cases, medications taken by patients around the time of liver sampling were cross-referenced with the LiverTox.nih.gov database to identify probable agents of drug-induced liver injury.^11^ The possible offending agents included ipilimumab (in 3 patients), darunavir/cobicistat/emtricitabine/tenofovir alafenamide (Symtuza), doxycycline, fluconazole, leflunomide, etanercept, phenytoin, divalproex, duloxetine (in 2 patients), gabapentin, celecoxib, tetracycline, and turmeric.

### Real-world utilization of molecular testing

Of the 251 cases of liver granulomas at the University of Utah, 2 were sent to an outside reference laboratory for pathogen molecular testing. In the first case, *C albicans* was identified by panfungal sequencing after GMS staining confirmed the presence of fungal hyphae (see **Figure 2 H**). The second instance was performed for a case with necrotizing granulomas, ultimately classified as idiopathic after molecular testing (broad-range bacterial, mycobacterial, and panfungal sequencing) and other workup were unrevealing; of note, this was not one of the 7 cases we found “highly suspicious for infection” in our retrospective review (see “Idiopathic Cases” above).

### Diagnostic yield of molecular testing in preselected cohorts

#### University of Utah

We collected data on any cases (not limited to liver specimens or specimens containing granulomas) at the University of Utah that underwent send-out pathogen molecular testing. Ninety-one specimens were sent over a 6-year period (2019-2024); organisms were detected in 49 cases (54%), while testing was negative in 42 cases (46%) (see **Table 4**). Twenty-three of the 49 positive cases (47%) were determined to be false positives based on clinicopathologic correlation and therefore treated as negative for infection. Nine of the 42 negative cases (21%) were determined to be false negatives, and the patients were treated for infection. The indications for molecular testing were categorized as “low clinicopathologic suspicion for infection” (35 cases), “high clinicopathologic suspicion for infection” (37 cases), and “species identification of cases with histologic evidence of organisms” (19 cases). Molecular testing helped to confirm infection in 3% (1/35), 32% (12/37), and 68% (13/19) of cases, respectively. These rates were determined after accounting for false-positive results in 92% (12/13), 43% (9/21), and 13% (2/15) of cases, respectively. Causes of false-positive results included contaminant bacteria (91%, 21/23) such as *Cutibacterium acnes*, *Methylorubrum* spp, and *Rhodococcus* spp; fungi (26%, 6/23) such as *Malassezia restricta* and *Knufia epidermidis*; and mycobacteria (9%, 2/23) such as *Mycobacterium avium*. Out of the 26 true-positive results, 16 (62%) included bacteria, 8 (31%) included mycobacteria, and 3 (12%) included fungi.

**Table 4:** Yield of Molecular Testing on Specimens From the University of Utah^a^

Characteristic	Positive (total), No.	Positive (false), No.	Positive (true), No.	Negative (total), No.	Negative (false), No.	Negative (true), No.
Low suspicion for infection	13	12	1	22	0	22
High suspicion for infection	21	9	12	16	5	11
Species-level identification of organisms seen histologically	15	2	13	4	4	0

a Among 91 recent specimens sent for molecular testing, 35 had low suspicion for infection, 37 had high suspicion for infection, and 19 were sent for species identification of organisms visualized histologically.

#### Washington University in St Louis

We collaborated with Washington University in St Louis to expand our data on the yield of send-out pathogen molecular testing in specimens suspected of harboring an infectious organism. Among 11 liver specimens sent for pathogen molecular testing over a 6-year period (2017-2022), organism detection occurred in 7 cases, while 4 were negative (see **Table 5**). One of the 7 positive results was considered a false positive, and there were no false negatives. The indications for molecular testing were similarly categorized as “low clinicopathologic suspicion for infection” (1 case, considered a true negative), “high clinicopathologic suspicion for infection” (8 cases, 4 considered true positives), and “species identification of cases with histologic evidence of organisms” (2 cases, both considered true positives). Organisms identified by molecular testing included bacteria (*C acnes*, *Morganella morganii*, *Fusobacterium nucleatum*), fungi (*C albicans*, *Saccharomyces cerevisiae*), and a parasite (*Schistosoma mansoni*). The single false-positive case identified *Mycobacterium fortuitum* and multiple other bacteria.

**Table 5:** Yield of Molecular Testing on Liver Specimens From Washington University in St Louis^a^

Case No.	Indication for molecular testing	Result	Clinical presentation	Ancillary pathology testing	Follow-up
1	Low suspicion for infection	Negative	History of autoimmune hepatitis and metabolic dysfunction-associated steatohepatitis following liver transplant. Presented with elevated aminotransferases concerning for rejection. Liver biopsy specimen showed noncaseating granulomas. Treated with steroids.	AFB (–) GMS (–) CMV (–) *T pall* (–) EBER (–) Adeno (–)	Infection was ruled out. Aminotransferases returned to baseline levels.
2	High suspicion for infection	Negative	Presented with acute right abdominal pain, emesis, and dark urine. Imaging showed a liver lesion. Liver biopsy specimen showed necrotizing granulomas. Treated with outpatient antibiotics.	AFB (–) GMS (–) Fite (–) *Bartonella* PCR (–)	Resolution of liver lesion on repeat ultrasound. No further treatment indicated.
3	High suspicion for infection	Negative	History of cystic fibrosis following lung transplant, complicated by aspergillosis. Presented with new ascites with elevated bilirubin and alkaline phosphatase. Blood culture was positive for *Enterococcus faecium* and *Escherichia coli*. Treated with antibiotics.	GMS (–) CMV (–) Gram (+ for focal bacteria)	Treated additionally with ursodiol. Deceased due to severe septic shock, prior to molecular results.
4	High suspicion for infection	Negative	History of ampullary carcinoma following Whipple procedure. Presented with abdominal pain and fever. Imaging showed multiple liver lesions. Initial liver biopsy specimen showed necrotizing granulomas. Treated with antibiotics. Repeat biopsy specimen showed reactive inflammatory changes without granulomas.	AFB (–) GMS (–)	Biliary strictures were identified, explaining the patient’s presentation of cholangitis.
5	High suspicion for infection	False positive (multiple bacteria, *Mycobacteria fortuitum*)	History of pancreatic cancer after Whipple procedure, followed by a recent hospitalization for acute cholangitis and melena. Presented with an enlarging peripheral liver lesion, previously biopsied to show granulomatous inflammation. Blood culture was positive for *Klebsiella* spp.	AFB (–) GMS (–)	Since the patient appeared relatively well, the organisms identified on molecular testing were considered contaminants. Other causes (autoimmune, rheumatologic) were pursued.
6	High suspicion for infection	Positive (*Cutibacterium acnes*)	Presented with severe right upper quadrant pain. Workup showed a liver lesion on imaging and positive *Bartonella* spp serology. After treatment for cat scratch disease, there was recurrent worsening pain. Liver biopsy specimen showed necrotizing granulomas.	AFB (–) GMS (–) Fite (–) WS (–) *Bartonella* PCR (–)	No further documentation of follow-up.
7	High suspicion for infection	Positive (*C acnes*, *Saccharomyces cerevisiae*)	History of nonseminomatous germ cell tumor following stem cell transplant. Presented with fever and liver lesions on imaging. Molecular testing was requested by the clinician. Treated with antibiotics and antifungals.	AFB (–) GMS (–) PAS (–)	Resolution of liver lesions. Was stable enough to receive second stem cell transplant.
8	High suspicion for infection	Positive (*Morganella morganii*)	History of acute myeloid leukemia following 2 stem cell transplants. Presented with sepsis and multiple hepatic abscesses. Treated empirically with antifungals. Blood culture was positive for *Morganella* spp.	AFB (–) GMS (–) WS (–) Gram (–)	Treated with antibiotics. Patient became stable with negative cultures.
9	High suspicion for infection	Positive (*Fusobacterium nucleatum*)	Presented with altered mental status, with brain and liver lesions on imaging. Liver function tests were within normal limits. Molecular testing was requested by the clinician. Treated with empiric antibiotics.	AFB (–) GMS (–) *T pall* (–)	Treated with antibiotics administered by image guidance.
10	Species-level identification of organisms seen histologically	Positive (*Schistosoma mansoni*)	History of immigration from Ethiopia and tuberculosis. Presented with fever, myalgia, diarrhea, and jaundice. Treated initially with antimycobacterial drugs. Liver biopsy specimen showed *Schistosoma* organisms, with positive serology.	AFB (–) GMS (–) PAS (–) EBER (–)	Discontinued antimycobacterial drugs. Treated with ursodiol and antiparasitic drugs.
11	Species-level identification of organisms seen histologically	Positive (*Candida albicans*)	History of 2 liver transplants. Presented with acute liver failure. Liver biopsy specimen with GMS stain showed fungal organisms, with blood cultures positive for *C albicans*. Treated with antifungals.	AFB (–) GMS (+ for angioinvasive fungus)	Patient deceased prior to molecular results.

Abbreviations: Adeno, adenovirus; AFB, acid-fast bacilli; CMV, cytomegalovirus; EBER, Epstein-Barr encoding region; GMS, Grocott’s methenamine silver; PAS, periodic acid–Schiff; PCR, polymerase chain reaction; *T pall*, *Treponema pallidum*; WS, Warthin-Starry; +, positive; –, negative.

a Among 11 specimens sent for molecular testing, 1 had low suspicion for infection, 5 had high suspicion for infection, and 2 were sent for species identification of organisms visualized histologically.

## DISCUSSION

### Causes and demographics of hepatic granulomas

In total, 251 cases of liver granulomas were identified at the University of Utah over a 16-year period. Overall, there was a small, insignificant difference in the causes between immunocompromised and immunocompetent populations. Most liver granulomas were idiopathic and comprised 31% of cases, a proportion similar to previously reported data in the literature.^4^^,^^5^ Ten percent of liver granulomas were attributable to sarcoidosis compared with proportions of around 3% to 5% in most other studies worldwide.^12‐14^ When excluding lipogranulomas, 12% of liver granulomas in our dataset were explained by sarcoidosis compared with 8% in another study.^5^ This difference in prevalence may be explained by the epidemiology of disease. Indeed, some causes are more common in certain geographic locations.^4^ For example, a study in Iran showed that most liver granulomas were attributed to infection by tuberculosis (53%), followed by visceral leishmaniasis (8%).^12^ A study in Greece showed that most liver granulomas were attributed to autoimmune disease (68%), followed by sarcoidosis (7.5%).^15^ On the other hand, a study in the United States with 88 cases showed that most liver granulomas were attributed to sarcoidosis (22%), in contrast to other parts of the world.^9^ A similar abundance of sarcoidosis diagnoses in our dataset prompted us to rereview and perform infectious stains on all cases. All cases were negative for infection by a limited panel of histochemical stains, and most cases were morphologically and clinically consistent with sarcoidosis. One case demonstrated a single nonnecrotizing poorly formed portal granuloma in a patient with a self-reported history of pulmonary sarcoidosis who had stopped treatment with prednisone because of adverse effects. In this scenario, the diagnosis remains uncertain given the absence of mediastinal or pulmonary disease on imaging. Sarcoidosis is a diagnosis of exclusion that relies on 3 general principles: compatible clinical and radiologic findings (pulmonary nodules, bilateral hilar lymphadenopathy, uveitis, fever), biopsy showing noncaseating granulomas in multiple organ systems, and exclusion of other causes of granulomatous disease.^16^ In the absence of classic histologic and clinical findings, the diagnosis of sarcoidosis based on hepatic granulomata may not be assumed, and other causes should be entertained and excluded.

Three other cases showed classic histology for sarcoidosis, but no evidence of pulmonary disease was found. Sarcoidosis is a systemic disease, so disease isolated to the liver (especially without pulmonary findings) would be a highly unusual presentation.^17^ These clinical scenarios highlight the caution needed in raising the possibility of sarcoidosis, given its lifelong clinical and therapeutic implications. For example, if a single well-formed nonnecrotizing granuloma is identified in a patient without a clear clinical cause, sarcoidosis may be considered, but the pathologist may want to emphasize that the finding is nonspecific and that the differential includes other causes (ie, drug reaction). On the other hand, our data included 2 cases where the identification of incidental liver granulomas prompted evaluation for pulmonary disease, leading to the diagnosis of sarcoidosis. Ultimately, the primary role of the pathologist is to report histologic findings, provide a differential diagnosis, and recommend clinicopathologic correlation, allowing the clinician to integrate the findings into their comprehensive assessment of the patient’s clinical presentation.

Our study highlights the important role of pathology in determining the cause of hepatic granulomata (see **Table 1**). For etiologies such as steatohepatitis and foreign body reaction, histopathology formed the basis for diagnosis and was therefore indispensable in all cases. For drug-induced liver granulomas, on the other hand, the pathologist could sometimes favor a drug as the offending agent and rule out other causes; however, the diagnosis would more often be made by the clinical team, who is patient-facing, with access to the patient’s full clinical picture. For liver granulomas attributed to sarcoidosis, the pathologist played a pivotal role in 38% of cases; this number would likely be higher if most patients had not already received a sarcoidosis diagnosis from prior lung and lymph node biopsy specimens. For liver granulomas attributed to infection, histopathologic findings were relied on in 45% of cases. Infectious organisms are often elusive on hematoxylin and eosin or special stains and may be detected by culture or serologic tests; when all of these modalities are uninformative, molecular testing can enhance the detection of infections.

### Real-world utilization gaps in molecular testing

A significant subset (9%) of the idiopathic cases demonstrated patterns of injury that were highly suspicious for infection (see **Figure 1** and **Table 2**). Subsequent histochemical staining and cultures were unrevealing, and no further infectious workup or treatment was attempted clinically, with varying patient outcomes (3 cases of complete resolution of symptoms/findings, 2 cases of death soon after biopsy, and 2 cases without documentation).

The most elemental workup for hepatic granulomas includes GMS and AFB special stains for fungal elements and acid-fast bacilli, respectively. The AFB stain has a reported sensitivity of around 37%,^18^^,^^19^ whereas GMS has high sensitivity for detecting fungal elements but low specificity for differentiating fungal genera.^20^ However, even if fresh tissue is obtained, cultures may be ineffective for detecting fastidious organisms such as *Bartonella* spp, *Neisseria* spp, *Legionella* spp, and *Bordetella* spp.^21^ Thus, special stains and tissue cultures alone may not constitute a sufficient workup when an infectious cause is suspected. Our study supports considering Epstein-Barr virus and syphilis in the differential when evaluating hepatic granulomata. In addition, our findings may support considering further molecular testing, particularly for fastidious bacterial pathogens. Potential barriers exist, including cost. Whereas histochemical stains typically cost under $100 each, molecular testing can cost hundreds to thousands of dollars per test. The yield of panfungal sequencing is low when no organisms are identified by stains, and there is a high risk of contamination, leading to false-positive identification^22^^,^^23^; thus, molecular testing for fungal pathogens is not recommended when fungal elements are not identified in tissue. However, when bacterial infections are in the differential, the yield of molecular testing, including sequencing over special and immunohistochemical stains, is often higher, so bacterial and mycobacterial molecular testing should be considered for suspicious cases even if stains and cultures are negative.^24^^,^^25^

Commercial molecular testing for pathogens was not widely available until the early 2000s, limiting the scope of older retrospective studies performed in the United States.^2^^,^^9^^,^^10^ However, among our 251 cases of liver granulomas from 2009 to 2024, only 2 underwent send-out molecular testing for pathogens. The first instance confirmed a known organism (1 of 20 infectious agents identified in our study). The other case remained idiopathic after negative results on molecular testing. Thus, molecular testing may be warranted for more cases, particularly for the 9% of idiopathic cases that were highly suspicious for infection. Potential barriers to molecular testing include cost (as discussed above), lack of experience with test ordering, excessive trust in conventional workup methodologies (ie, histology, stains, culture, serology), and the additional time and effort required to order a send-out test at an outside facility. However, with the widespread availability of molecular testing nowadays, pathologists must be more proactive in using this resource.

### Diagnostic yield of molecular testing in preselected cohorts

Given the historical scarcity of liver specimens from the University of Utah that were sent for broad-range pathogen molecular sequencing, we evaluated molecular data on all specimens (n = 91) to establish baseline assay performance and false-positive contamination rates in formalin-fixed, paraffin-embedded specimens. These data directly complement and contextualize the cohort from Washington University in St Louis (n = 11), which examined molecular data on liver specimens only. The results from both cohorts demonstrated consistent diagnostic trends across specimen types.

#### University of Utah

Ninety-one specimens at the University of Utah in a 6-year period (2019-2024) were sent for pathogen molecular testing. The indications for testing were categorized as “low clinicopathologic suspicion for infection,” “high clinicopathologic suspicion for infection,” and “species identification of cases with histologic evidence of organisms.” The increasing levels of suspicion for infection correlated positively with rates of successful pathogen identification by molecular testing (2.9%, 32.4%, and 68.4% respectively). These statistics demonstrate the high yield of molecular testing when suspicion for infection is high, as well as occasional diagnostic utility even when suspicion is low.

The above rates also accounted for false-positive results (ie, contaminants), whose frequencies were inversely correlated with the level of suspicion for infection (92.3%, 42.9%, and 13.3%, respectively). Most contaminants consisted of bacteria (91.3%) and fungi (26.1%), not mycobacteria (8.7%). In all cases, the distinction between contaminants and true pathogenic infection was straightforward and determined based on the variety of organisms (the presence of 3 + species favors contamination), whether they are commonly found on the skin/environment, organism abundance (trace or smaller amounts favor contamination), and correlation with clinical and pathologic findings. In several cases, pathogenic agents were singled out from a background of contaminants without significant difficulty.

Among the true pathogens identified, 62% were bacteria, 31% were mycobacteria, and 12% were fungi. These findings suggest that the yield of molecular testing is greater for bacteria and mycobacteria than for fungi, though several preanalytic factors should be acknowledged. Epidemiologically, bacterial infections are more prevalent than fungal infections (although there may be variation by region), so pathogen identification may be skewed toward bacteria.^26^ Clinically, the utility of molecular testing also varies by pathogen type. For example, testing for mycobacterial infection and confirming the species of mycobacteria are often critical because of the potential severity of disease as well as the morbidity and specificity of treatment regimens.^27^^,^^28^ Identification of mycobacteria with other methods (ie, culture) can also be ineffective and slow because of their fastidious nature.^29^ On the other hand, bacteria and fungi are usually more easily identified by culture and are often initially treated with empiric antimicrobial drugs. These considerations help to explain the overrepresentation of mycobacteria in pathogen molecular testing.

A review of send-out cases highlighted numerous other benefits of molecular testing. For example, positive identification can guide antimicrobial coverage, while negative results can facilitate other treatments (ie, corticosteroids to reduce cerebral edema) and prompt workup for noninfectious causes. Molecular testing is particularly useful for ruling out specific organisms (ie, in cases of suspected recurrent or residual infection or spread of a known infection to a different site). For instance, in 1 case, molecular studies identified recurrent/residual *Escherichia coli* in a neonate despite negative cultures following a completed course of antibiotics. This finding provided clinical and prognostic information: while late-onset infection of hospitalized premature neonates by gram-positive organisms is relatively common and has low mortality, infection by gram-negative coliform bacteria carries a high mortality risk.^30^ Finally, the robustness of molecular testing makes it useful in scenarios where culture material cannot be obtained, such as in autopsies. The identification of a pathogen that resulted in fetal demise can provide insight into the source of infection and help determine risk factors to avoid in future pregnancies. Even with these benefits, false-positive and false-negative results are a concern. In cases where infection was highly suspected, negative molecular results were considered spurious and were attributed to sampling error, specimen degradation (most specimens were formalin fixed), or antimicrobial administration prior to specimen collection.

#### Washington University in St Louis

Eleven liver specimens evaluated at Washington University in St Louis in a 6-year period (2017-2022) were submitted for send-out pathogen molecular testing. Pathogens were identified in 0% of cases with “low clinicopathologic suspicion for infection,” 50% of cases with “high clinicopathologic suspicion for infection,” and 100% of cases with “histologic evidence of organisms.” These data follow a trend similar to those from the University of Utah (see section above), again demonstrating the potential value of molecular testing when there is high suspicion for infection. In all cases, molecular testing provided information useful for therapeutic guidance or ruling out infection. Notably, a parasite (*S mansoni*) was identified in 1 case by 28S ribosomal RNA gene sequencing. While the test has only been validated for fungal detection, the 28S ribosomal RNA gene is present in all eukaryotic organisms, allowing for incidental detection of some parasites.^31^

### Study limitations

This study has several limitations. First, our evaluation of molecular diagnostic yield is inherently subject to selection bias. Because broad-range pathogen sequencing was requested at the discretion of treating clinicians and pathologists rather than applied unselectively across all granulomatous liver biopsy specimens, the tested subset represents a preselected cohort with a high prior probability of an infectious cause. Nevertheless, these highly suspicious cases may benefit most from molecular testing. Second, broad-range molecular data were acquired from a heterogeneous set of specimens at the University of Utah and from a relatively small sample size at Washington University in St Louis, limiting our ability to make broad statistical claims about diagnostic yield.

Stratification of the data by immune status was a main goal in this project. However, determination of immune status relies on comprehensive and accurate medical records, which may be unreliable, especially in older records, where there was often little to no patient health information available. A similar dilemma exists for cataloging medications responsible for drug-induced liver granulomas. Drug exposure information in the electronic medical record may omit supplements or other over-the-counter medications, including those taken sporadically. Finally, the primary data collection was performed at a single institution retrospectively. This is both a weakness as well as a strength: while our data may not be applicable to populations in other regions, they provide a useful update on the causes of liver granulomas in the United States.

## CONCLUSION

The purpose of this study was to explore the causes of hepatic granulomata in a large tertiary center in the United States and compare the findings with prior data. Upon stratifying patients by immune status, some infections (mycobacteria) were seen preferentially in immunocompromised patients, while others (hepatitis C virus, *C burnetti*, *H capsulatum*) were seen in immunocompetent patients. The study reveals the possible underuse of molecular testing in cases highly suspicious for an infectious cause when stains and cultures are unrevealing. Interestingly, the study also highlighted the importance of clarity in communicating the possible diagnosis of sarcoidosis. Overall, pathologists are particularly impactful in the workup and diagnosis of liver granulomata for both infectious and noninfectious diseases, and the findings communicated by pathologists in their reports have significant implications in clinical decision-making. As key members of the patient’s care team, pathologists have the unique and important role of facilitating additional ancillary testing for infectious agents.

## Data Availability

The data underlying this article will be shared on reasonable request to the corresponding author.
